# Pituitary Macroadenoma Secondary to Congenital Hypothyroidism With Growth Failure and Developmental Delay: A Rare Presentation

**DOI:** 10.7759/cureus.39655

**Published:** 2023-05-29

**Authors:** Sham Lohiya, Chitturi Venkata Sai Akhil, Shubhangi Patil Ganvir, Richa Chaudhary, Jayant Vagha

**Affiliations:** 1 Department of Pediatrics, Jawaharlal Nehru Medical College, Datta Meghe Institute of Medical Sciences, Wardha, IND

**Keywords:** congenital hypothyroidism, pituitary macroadenoma, hypothyroidism, growth, pituitary hyperplasia, pituitary adenoma, levothyroxine

## Abstract

Reactive pituitary hyperplasia can develop as a pituitary (pseudo) macroadenoma in the case of primary hypothyroidism. Hypothyroidism-induced pituitary hyperplasia (PHPH) can be managed medically. Surgery should not be performed if this condition is misdiagnosed as an adenoma. Primary hypothyroidism is a well-known cause of children's slow linear growth. Anterior pituitary enlargement is a rare symptom of severe or long-term illness (pituitary pseudotumor). Thyroid-stimulating hormone-secreting adenomas (TSHomas) are the rarest type of pituitary adenomas, with most endocrinologists seeing just a few cases throughout their lives. In most situations, the diagnosis is difficult, and patients may be referred after presenting with a condition of excessive thyroid-stimulating hormone secretion or a pituitary tumor.

In this case study, we describe a 3.5-year-old girl who was referred to our hospital for a surgical assessment of a suspected pituitary neoplastic lesion. It was later determined that the suspected lesion was really pituitary hyperplasia brought on by primary hypothyroidism. Levothyroxine was started, and the dose was increased. The patient was advised to follow up to see if pituitary macroadenoma had responded to levothyroxine supplementation.

Pituitary enlargement (pseudotumor of the pituitary gland) is a rare complication of primary hypothyroidism. Early diagnosis and treatment are critical for children with severe primary hypothyroidism to maintain their final height, as late diagnosis nearly always leads to a decline in adult stature. Pituitary macroadenoma secondary to severe hypothyroidism does not need risky and expensive surgical intervention. Because PHPH is rare in children, more credible information is needed to have a better knowledge of how the disease progresses and to develop scientific diagnostic criteria.

## Introduction

Hypothyroidism is a well-known cause of children's poor linear growth. Pituitary enlargement (pituitary pseudotumor) can occur when hypothyroidism is persistent or severe. Pituitary hyperplasia (PH) can occur as a result of excessive thyrotropin-releasing hormone (TRH) levels, which stimulate pituitary thyrotroph cells, causing the pituitary gland to enlarge [1]. The pituitary gland can become noticeably enlarged in some patients when brain magnetic resonance imaging (MRI) is performed prior to thyroid tests, and a macroadenoma may be included in the differential diagnosis [2]. Pituitary macroadenomas are uncommon in children and are notoriously difficult to diagnose. They manifest both endocrine and neurological signs and symptoms. Endocrine symptoms are due to pituitary hormone dysfunction, whereas neurological symptoms are caused by the mass effect and can emerge after endocrine changes and growth retardation [3]. Thus, if a pituitary macroadenoma is suspected, a thorough endocrine examination is required to avoid expensive and risky surgical treatments.

Pregnancy, puberty, and endocrine disorders are all examples of physiological and pathological events that can cause pituitary hyperplasia [3]. Niepce noted the growth of the sella turcica in cretins with hypothyroidism in 1851, and this was the first description of pituitary hyperplasia following primary hypothyroidism [4]. Although this illness was previously thought to be unusual, it has recently received extensive reporting in the literature. Pituitary hyperplasia as a result of primary hypothyroidism, on the other hand, is uncommon in children. Unnecessary neurosurgery procedures could be avoided with a proper diagnosis.

In this case study, we describe a 3.5-year-old girl who was referred to our hospital for a surgical assessment of a suspected pituitary neoplastic lesion. It was later determined that the suspected lesion was really pituitary hyperplasia brought on by primary hypothyroidism.

## Case presentation

**Figure 1:**
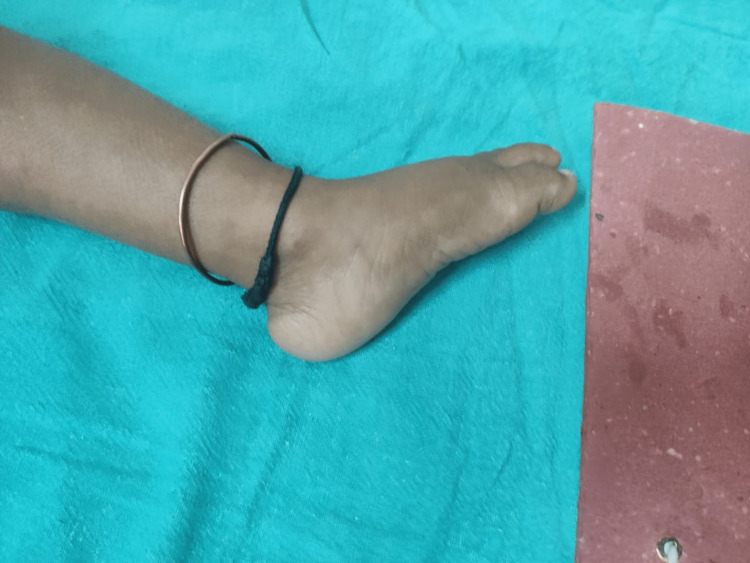
Rocker bottom foot

A 3.5-year-old female child was brought with concerns of not being able to walk since inception. This was associated with complaints of the baby being floppy and hypotonic in nature. No other history of any known illness or of previous hospitalization was given apart from the baby being admitted to a NICU on the third day of life for jaundice and apparently requiring a blood transfusion, as narrated by the mother. The mother also had a history of first-trimester abortion and was going on to need medical attention for secondary infertility. No history of any known disease in the parents or anyone else in the family was found. The child had a global development delay with only being able to sit with support, cooing occasionally, only being able to scribble if handed a pen, having evident stranger anxiety, and not being able to wave goodbye. On physical examination, the patient had coarse facies, flat feet, almost rocker bottom feet (Figure 1), and a typical screeching hoarse cry.

**Figure 2:**
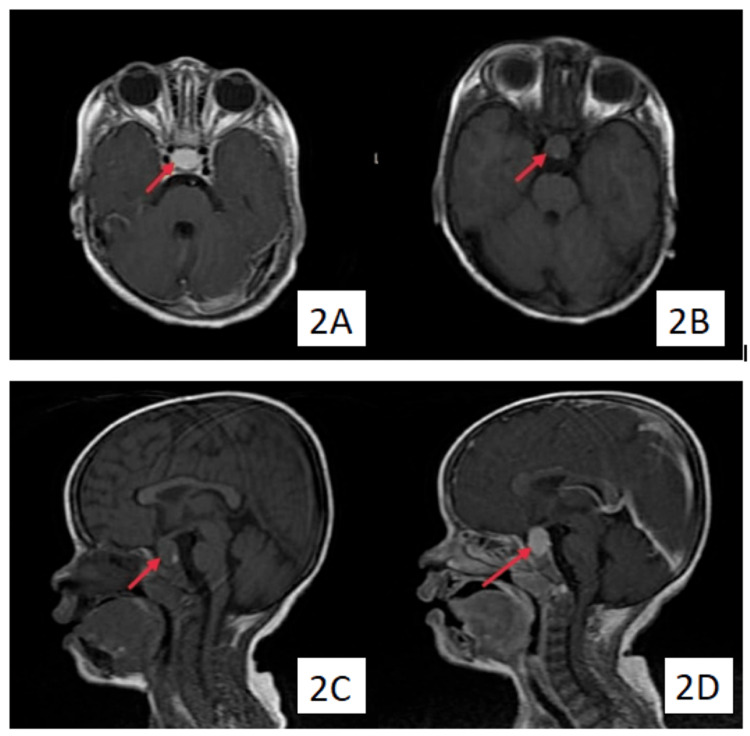
(A) Axial T1-weighted (post-contrast), (B) axial T1-weighted (pre-contrast), (C) coronal T1-weighted (pre-contrast), and (D) coronal T1-weighted (post-contrast) sections showing a homogenously enhancing extra-axial mass lesion in the sellar and suprasellar lesion

She weighed about 10 kg and had only achieved a height of 78 cm at 3.5 years of age with a head circumference of only 45 cm, all well below the third centile for the given age and sex. The child upper segment to lower segment ratio was 1.29 with midparental height being 148 cm. The patient was evaluated routinely along with a complete thyroid panel. An MRI of the brain with gadolinium contrast was also done as an adjunct to rule out common causes of developmental delay in a private clinic. MRI of the brain suggested a homogenously enhancing extra-axial mass lesion noted in the sellar and suprasellar lesion measuring about 16 x 11 x 11 mm, which was causing widening of the sella, giving it a figure of 8 appearance. This lesion was also reported to cause a mass effect, abutting the adjacent carotid artery laterally and the optic chiasma superiorly, likely to be a pituitary macroadenoma (Figure 2). The patient was referred to our institution for neurosurgical intervention. While other routine workups showed relatively normal values, a thyroid profile was done as a part of the workup for poor growth and developmental delay, which showed ceiling-high thyroid-stimulating hormone (TSH) levels (>1200 microIU/ml). Hung up reflex was present on examination. An ophthalmological evaluation was done to assess the field of vision, but the child was not cooperative for perimetry.

Hence, pituitary macroadenoma was presumed to be secondary to severe hypothyroidism. The patient was started on levothyroxine 25 mcg/day (2.5 mcg/kg/day) and the dose was increased to 50 mcg/day (5 mcg/kg/day) after seven days. An improvement in the mental status was observed in the child after starting levothyroxine. The patient was discharged and was advised for a follow-up MRI of the brain after two months to see if pituitary macroadenoma is responding to thyroid supplementation.

## Discussion

Primary hypothyroidism in children is characterized by a variety of symptoms and signs, the most common of which are a slowing of height growth and an increase in body weight [5]. Because MRI is becoming more widely available, brain imaging is being used at an earlier stage of the diagnostic workup for delayed growth. Reactive pituitary hyperplasia is not uncommon in severe primary hypothyroidism, although the differential diagnosis might be difficult due to the imaging similarities between pituitary macroadenoma and severe pituitary hyperplasia [6]. Pituitary hyperplasia brought on by hypothyroidism may be misdiagnosed as a macroadenoma if an MRI is inappropriately done prior to thyroid function tests.

Desai et al. in 1996 reported the first case of hypothyroidism in children linked to pituitary enlargement [7]. Low thyroid hormone levels in primary hypothyroidism reduce the thyroid hormone's negative feedback to the hypothalamus, causing oversecretion of TRH and proliferation of TSH-secreting cells, resulting in hypothyroidism-induced pituitary hyperplasia (PHPH) [8]. The most prevalent cause of hypothyroidism in children and teenagers is Hashimoto's thyroiditis. Based on laboratory results, imaging results, and clinical signs, it can be difficult to distinguish PHPH from a pituitary adenoma. Accurate identification is essential because PHPH and pituitary adenoma have very distinct treatments. Patients with pituitary adenoma are often treated with surgical resection, but those with pituitary hyperplasia benefit from thyroid hormone replacement therapy [9]. In the event of pituitary hyperplasia, surgical removal of the pituitary would result in irreversible pituitary dysfunction as well as growth and mental retardation in children.

The clinical manifestations of PHPH include symptoms of hypothyroidism (fatigue, cold intolerance, myxedema, etc.), menstrual disorders, galactorrhea, and infertility [10]. Growth retardation and obesity are the most common symptoms of PHPH, which can also be accompanied by hypothyroidism, early puberty, and hyperprolactinemia. PHPH seldom impairs a child's intelligence quotient (IQ) [10].

Pituitary hyperplasia following primary hypothyroidism in children is characterized by hypothyroidism-related symptoms, such as short stature, tiredness, and myxedema, as well as prolactin-related symptoms, such as menstruation problems and galactorrhea [11]. In children, visual impairment and neurological disorders are uncommon. For decades, pituitary hyperplasia has been diagnosed using CT and MRI, both with and without contrast [12]. The pituitary gland is normally seen as a homogeneous, isodense, diffuse, and symmetrical enlargement on MRI. In the midline sellar area, a CT depicts a spherical, isodense mass with homogeneous enhancement [13]. Our findings are consistent with what has been reported in the literature.

Sellar masses in children can be caused by a variety of conditions, such as craniopharyngeal neoplasms, intracranial germ cell tumors, pituitary adenoma, and pituitary hyperplasia [14]. Pituitary adenoma and hyperplasia have comparable radiological and clinical characteristics, making it difficult to distinguish between the two. Although pituitary adenoma has been recorded in the context of primary hypothyroidism, it is extremely uncommon. Given the pituitary mass and elevated TSH level, a thyrotropin-producing pituitary adenoma may be a possibility, although these tumors are extremely rare, accounting for only 0.5% of all pituitary adenomas [15]. The most common cause of hypothyroidism in children that leads to pituitary hyperplasia is Hashimoto's thyroiditis. Subtotal thyroidectomy, radioactive iodine therapy, and medications like interferon and thionamides are other common causes [16]. None of these drugs were being used by our patient.

Although there are no obvious signs of hemorrhage, necrosis, or cystic fibrosis, intracranial MRI imaging demonstrates diffuse development of the anterior lobe of the pituitary gland with homogenous T1 and T2 signals. A dome-shaped blunt edge change might be seen as a sign of sellar extension. The extension is uniformly enhanced, with a peak enhancement that is comparable to that of the normal pituitary gland. The posterior lobe of the pituitary gland has a high signal intensity in normal circumstances. The pituitary stalk may become normal or thickened but not distorted as the disease progresses, with a suprasellar extension forming a gourd-shaped appearance, most likely extending on the suprasellar cistern or occasionally compressing the optic chiasm, but rarely invading the cavernous sinus or skull bone [17].

Following treatment with levothyroxine, the size of the pituitary gland had been reported to be normalized on subsequent imaging scans. However, rather than starting at the full anticipated replacement dose, it may be preferable to gradually raise the amount of levothyroxine over time in individuals with severe clinical signs of hypothyroidism [17]. This is due to a variety of factors. First, patients with hypothyroidism-related myxedema and pericardial effusion may not be able to tolerate rapid euthyroidism, which could exacerbate heart failure symptoms. Since young children who are generally healthy can tolerate rapid thyroid hormone replacement, this is mostly an issue for adults or children who have other comorbidities. Second, it has been noted that in severe, long-term primary hypothyroidism, pituitary and adrenal function may be decreased secondary to hypothyroidism, and adrenal insufficiency may be precipitated by rapid thyroid hormone replacement. Third, there have been case reports of patients with thyrotroph hyperplasia due to primary hypothyroidism who acquired an empty sella after receiving levothyroxine replacement medication, possibly indicating that rapid reduction of the enlarged anterior pituitary caused pituitary apoplexy. Finally, pseudotumor cerebri has been identified as a probable side effect of levothyroxine therapy.

## Conclusions

From this case, we infer that early diagnosis and treatment are critical for children with severe primary hypothyroidism to maintain their final height, as late diagnosis nearly always leads to a decline in adult stature. Because PHPH is not common in children, more credible information is needed to have a better knowledge of how the disease progresses and to develop scientific diagnostic criteria. Even though thyroid hormone replacement therapy can improve these symptoms, some children with severe dysplasia or short stature still struggle to grow properly. Accurate diagnosis may save the patient from undergoing needless neurosurgical procedures. As a result, substantial study is required for early diagnosis of PHPH in children.
